# Panoramic Assessment of Mental Foramen Location in Adult Patients in Riyadh, Saudi Arabia: A Retrospective Cross-Sectional Study

**DOI:** 10.7759/cureus.72656

**Published:** 2024-10-29

**Authors:** Yahia Ahmad, Nadir Alajmy, Khalid Almobiedh, Abdulrahman Alsemari, Ghanem Alfailakawi, Soud Alenezi, Raviraj Jayam

**Affiliations:** 1 College of Dentistry Internship Department, Riyadh Elm University, Riyadh, SAU; 2 Oral and Maxillofacial Surgery and Diagnostic Sciences, Riyadh Elm University, Riyadh, SAU

**Keywords:** anatomy, localization, mandible, mental foramen, panoramic radiograph

## Abstract

Objectives: This retrospective cross-sectional study aimed to determine the localization of the mental foramen in a Saudi population using panoramic radiographs, identify the most common vertical and horizontal positions, assess bilateral symmetry, and analyze variations based on age and gender.

Methods: Digital panoramic radiographs of 504 patients, aged 18-58 years, were analyzed. The variables assessed included age group, gender, vertical position (Zones A, B, and C), horizontal position (Zones 1-6), and symmetry (present/absent) of the mental foramen location bilaterally. The mental foramen location was determined using standardized vertical and horizontal zones, and data were recorded on specialized sheets. Descriptive statistics, Pearson's chi-square test, and intra-examiner reliability were performed.

Results: The sample consisted of 250 (49.6%) females and 254 (50.4%) males. Vertically, Zone B coinciding with premolar apices was the most frequent position bilaterally, with 317 (62.9%) on the right side and 321 (63.7%) on the left side. Horizontally, Zone 4 between the first and second premolars was predominant, with 332 (65.9%) on the right side and 345 (68.5%) on the left side. High bilateral symmetry in the location was observed in 441 (87.5%) cases. Statistically significant differences were found in mental foramen localization based on gender (p < 0.05). No significant association was found between age and vertical position, although a tendency for more anterior horizontal positioning in older individuals was noted (p > 0.05).

Conclusion: Within the limitations of this panoramic assessment, the mental foramen was located most often inferior to premolar apices vertically and between the first and second premolars horizontally, with a high degree of bilateral symmetry. Gender had a significant impact on mental foramen position, while age showed a non-significant trend toward anterior horizontal localization. These findings guide dental procedures in the premolar region to avoid neurovascular damage.

## Introduction

The mental foramen (MF) is a bilateral opening in the buccal cortical plate of the mandible, which serves as the termination of the mandibular canal. It allows for the passage of the mental nerve, vein, and artery, which provides sensory innervation to the anterior buccal mucosa, lower lip, and skin of the chin region [1,2]. Accurate knowledge of the MF's location is crucial for avoiding injury to the mental nerve during various dental procedures, such as local anesthesia administration, surgical interventions, dental implant placement, and endodontic treatment in the mandible. However, locating the MF can be challenging as there are no absolute anatomical landmarks for reference, and the foramen cannot be clinically visualized or palpated. Consequently, radiographic imaging is essential for accurately determining the position of the MF [3].

In the past, numerous studies have been conducted to localize the precise position of the MF in different regions of Saudi Arabia. These studies employed various radiographic imaging techniques, such as orthopantomograms (OPGs), computerized tomography (CT), and cone-beam computed tomography (CBCT). According to Peker et al., OPGs have been reported to be sufficient for the accurate localization of the MF, with the added benefits of being easy to obtain, economical, and subjecting the individual to much less radiation compared to other imaging modalities [4,5]. However, these studies have reported different aims, sample sizes, methodologies, and results, making it difficult to draw definitive conclusions about the location of the MF in the Saudi population.

Factors such as age, gender, ethnicity, amount of ridge resorption, and tooth loss have been reported to contribute to the variation in the location of the MF [6,7]. For example, Ghandourah et al. examined the location of the MF in a sampled population of patients from four Umm Al-Qura University teaching hospitals, using 334 OPGs of both adult and pediatric patients aged 13 years and above. The study concluded that there was no significant difference in the location of the MF between genders [8]. In contrast, Mahabob et al., in a study conducted in the eastern province of Saudi Arabia, utilizing 101 CBCTs, concluded that the position of the MF varies according to gender, age, and ethnicity [9].

The rationale for this retrospective cross-sectional study is to accurately determine the most frequent location of the MF in the adult population of Riyadh, Saudi Arabia, utilizing precise measurements that combine both vertical and horizontal positioning methods. In this study, we aim to determine the most common vertical and horizontal location of the MF in the studied population, assess its symmetry between the right and left sides, and evaluate whether age and gender contribute to significant differences in its localization. By providing insights into these aspects, we strive to enhance the precision and safety of dental and surgical procedures involving the MF.

The hypotheses are that there is no significant difference in the vertical or horizontal location of the MF between the right and left sides; there is no significant association between age and MF localization; and there is no significant association between gender and MF localization. By accurately localizing the MF and analyzing symmetries and demographic patterns in this Saudi population, this study aims to provide clinically relevant data to guide effective dental procedures in the mandibular premolar region [10-18].

## Materials and methods

Study design and setting

This retrospective cross-sectional study was conducted to determine the localization of the MF in an adult Saudi population using panoramic radiographs. The research was carried out at three university dental hospitals (Al Munsiyah, Al Namudhajiyah, and Al Olaya) affiliated with Riyadh Elm University in Riyadh, Saudi Arabia. Ethical approval was obtained from the institutional review board before data collection.

Study duration

The study period spanned from June 21, 2009, to August 14, 2023, with the oldest radiograph dated June 21, 2009, and the most recent one dated August 14, 2023. The research work, including data analysis and manuscript preparation, was conducted between October 2023 and February 2024.

Participants and sample size

The target population consisted of adult patients aged 18-58 years who had undergone panoramic radiography during routine dental examinations at the study sites within the specified study period. Convenience sampling was utilized to select patients based on specific inclusion and exclusion criteria. Patients were included if they had complete bilateral premolar dentition and good-quality panoramic radiographs with visible anatomy in the mandibular premolar region. Patients were excluded if they had crowded or missing dentitions, bony lesions, a history of orthodontics, orthognathic surgery, dental implants, or medications affecting bone density in the studied region.

The minimum recommended sample size was calculated to be 377 panoramic radiographs based on calculations using Raosoft software (Raosoft Inc., Seattle, WA) with a 5% margin of error and 95% confidence level. After applying the selection criteria, a final sample of 504 radiographs was included in the study.

Variables and radiograph analysis

The key variables assessed in this study were age group (10-20, 21-30, 31-40, 41-50, and 51-60 years), gender (male/female), vertical position of the MF on the right and left sides (Zones A, B, and C), horizontal position of the MF on the right and left sides (Zones 1-6), and symmetry (present/absent).

Digital panoramic radiographs were retrieved from the hospital databases and analyzed using Sidexis XG version 2.61 software on an LCD monitor. The MF location was determined using standardized horizontal zones described by Al-Jasser and Nwoku [12] and vertical zones by Al-Juboori et al. [13]. The zonal location was recorded on specialized data collection sheets along with gender, age group, and symmetry. Intra-examiner reliability was verified by re-measuring 10% of the radiographs.

Statistical analysis

IBM SPSS Statistics version 25.0 (IBM Corp., Armonk, NY) was utilized for data analysis. Descriptive statistics were used to determine means, medians, standard deviations, and frequencies. Pearson's chi-square test was employed to evaluate correlations between MF localization and gender and age. A p-value < 0.05 was considered statistically significant.

In summary, this retrospective cross-sectional study analyzed 504 panoramic radiographs of adult Saudi patients aged 18-58 years, taken between June 21, 2009, and August 14, 2023, to determine the localization of the MF. The research work was conducted between October 2023 and February 2024. Standardized horizontal and vertical zoning systems were used to assess the MF position, and statistical analysis was performed to evaluate demographic variations and symmetry.

## Results

A total of 504 panoramic radiographs were included in this retrospective cross-sectional study after applying the pre-determined inclusion and exclusion criteria.

Table 1 shows a nearly equal representation of 254 males (50.4%) and 250 females (49.6%) in a sample of 504 individuals. The cumulative percentage reaches 100% for 250 females (49.6%), as it is the last category. The "total" row shows 504 (100.0%), matching the sum of frequencies for both genders, indicating no missing data.

**Table 1:** Gender distribution

Gender		Frequency	Percent	Valid Percent	Cumulative Percent
Valid	Male	254	50.4	50.4	50.4
	Female	250	49.6	49.6	100.0
	Total	504	100.0	100.0	100.0

**Figure 1:**
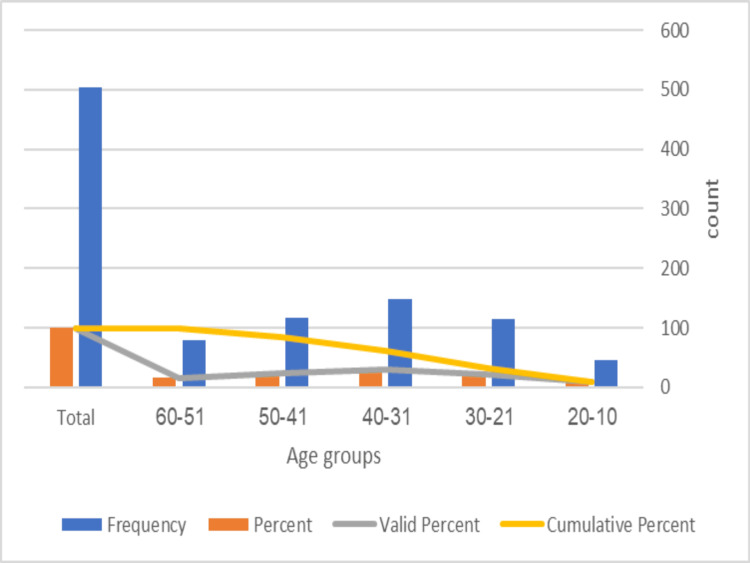
Age groups

Table 2 presents the age distribution of a sample of 504 individuals. The majority of the sample, comprising 148 individuals (29.4%), falls within the 31-40 age range, followed by those aged 41-50 years (116 individuals, 23.0%) and 21-30 years (114 individuals, 22.6%). The 51-60 age range accounts for 80 individuals (15.9%) of the sample, while the 10-20 age range has the lowest representation at 46 individuals (9.1%). Figure 1 illustrates the age groups.

**Table 2:** Age distribution

Age Range (Years)		Frequency	Percent	Valid Percent	Cumulative Percent
Valid	10-20	46	9.1	9.1	9.1
	21-30	114	22.6	22.6	31.7
	31-40	148	29.4	29.4	61.1
	41-50	116	23.0	23.0	84.1
	51-60	80	15.9	15.9	100.0
	Total	504	100.0	100.0	100.0

Localization of the MF was performed by dividing the anterior-posterior space from the mesial of the first mandibular molar to the canine into six horizontal zones, and the vertical space into three zones, for both left and right sides. Symmetry in location between sides was also analyzed. Quantitative data were recorded, and statistical analysis was carried out using SPSS version 25.0 (IBM Corp., Armonk, NY). Descriptive statistics are presented in Table 3.

**Table 3:** Descriptive statistics

Statistic	Mean	Median	Mode	Std. Deviation
Gender	1.5	1	1	0.5
Age	3.14	3	3	1.2
Vertical location zone (right)	2.34	2	2	0.504
Horizontal location zone (right)	4.08	4	4	0.671
Vertical location zone (left)	2.35	2	2	0.493
Horizontal location zone (left)	4.04	4	4	0.66
Symmetry	1.13	1	1	0.331

Table 3 presents descriptive statistics for various variables in a sample of 504 individuals. The gender distribution is balanced, with a mean of 1.50 and a median of 1.00. The mean age is 3.14, suggesting a relatively young sample, with the most common age group being 31-40 years (mode = 3). The vertical location zones on both the right and left sides have similar means (2.34 and 2.35) and medians (2.00), with the mode being 2. The horizontal location zones also show comparable means (4.08 and 4.04) and medians (4.00), with the mode being 4. The mean symmetry score is 1.13, with a median of 1.00 and a mode of 1, indicating a high degree of symmetry in the sample. The standard deviations for all variables are relatively low, suggesting minimal dispersion around the mean values.

Table 4 shows the distribution of the vertical location zone on the right side. The majority of the sample (317, 62.9%) falls within Zone B, followed by Zone C (180, 35.7%). Zone A has the lowest representation at 7 (1.4%). The cumulative percentage reaches 100% for Zone C (180, 35.7%), indicating no missing data. The data suggests that the vertical location on the right side is predominantly concentrated in Zones B and C, with minimal presence in Zone A.

**Table 4:** Vertical position frequency - right side

Vertical Location Zone (Right)		Frequency	Percent	Valid Percent	Cumulative Percent
Valid	A	7	1.4	1.4	1.4
	B	317	62.9	62.9	64.3
	C	180	35.7	35.7	100.0
	Total	504	100.0	100.0	100.0

Table 5 presents the distribution of the horizontal location zone on the right side. The majority of the sample (332, 65.9%) falls within Zone 4, followed by Zone 5 (106, 21.0%) and Zone 3 (50, 9.9%). Zones 1, 2, and 6 have minimal representation, collectively accounting for only 16 (3.2%) of the sample. The cumulative percentage reaches 100% for Zone 6 (2, 0.4%), indicating no missing data. The data suggests that the horizontal location on the right side is primarily concentrated in Zones 4 and 5.

**Table 5:** Horizontal position frequency - right side

Horizontal Location Zone (Right)		Frequency	Percent	Valid Percent	Cumulative Percent
Valid	1	2	.4	.4	.4
	2	9	1.8	1.8	2.2
	3	50	9.9	9.9	12.1
	4	332	65.9	65.9	78.0
	5	106	21.0	21.0	99.0
	6	5	1.0	1.0	100.0
	Total	504	100.0	100.0	100.0

Table 6 shows the distribution of the vertical location zone on the left side. The majority of the sample (321, 63.7%) falls within Zone B, followed by Zone C (179, 35.5%). Zone A has minimal representation at 4 (0.8%). The cumulative percentage reaches 100% for Zone C (179, 35.5%), indicating no missing data. The data suggests that the vertical location on the left side is primarily concentrated in Zones B and C, with a negligible presence in Zone A.

**Table 6:** Vertical position frequency - left side

Vertical Location Zone (Left)		Frequency	Percent	Valid Percent	Cumulative Percent
Valid	A	4	0.8	0.8	0.8
	B	321	63.7	63.7	64.5
	C	179	35.5	35.5	100.0
	Total	504	100.0	100.0	100.0

Table 7 presents the distribution of the horizontal location zone on the left side. The majority of the sample (345, 68.5%) falls within Zone 4, followed by Zone 5 (89, 17.7%) and Zone 3 (53, 10.5%). Zones 1, 2, and 6 have minimal representation, collectively accounting for only 17 (3.4%) of the sample. The cumulative percentage reaches 100% for Zone 6 (2, 0.4%), indicating no missing data. The data suggests that the horizontal location on the left side is primarily concentrated in Zones 4 and 5.

**Table 7:** Horizontal position frequency - left side

Horizontal Location Zone (Left)		Frequency	Percent	Valid Percent	Cumulative Percent
Valid	1	2	0.4	0.4	0.4
	2	10	2.0	2.0	2.4
	3	53	10.5	10.5	12.9
	4	345	68.5	68.5	81.3
	5	89	17.7	17.7	99.0
	6	5	1.0	1.0	100.0
	Total	504	100.0	100.0	100.0

Symmetry in the location of the MF between the right and left sides was observed in the majority of cases (Table 8). Table 8 shows the symmetry distribution in the sample. The vast majority, 441 (87.5%), of the sample exhibits symmetry, while only 63 (12.5%) do not. The cumulative percentage reaches 504 (100%) for the "No" category, indicating no missing data. The data suggests a high prevalence of symmetry within the sample.

**Table 8:** Symmetry frequency

Symmetry		Frequency	Percent	Valid Percent	Cumulative Percent
Valid	Yes	441	87.5	87.5	87.5
	No	63	12.5	12.5	100.0
	Total	504	100.0	100.0	100.0

The association between gender, age, and MF localization was assessed using cross-tabulation and chi-square tests. Table 9 presents the results of chi-squared tests for the association between gender and various location variables. The p-values for all location variables (right-side vertical, right-side horizontal, left-side vertical, and left-side horizontal) are less than 0.05, indicating statistically significant associations between gender and these variables. The strongest association is observed for the right-side horizontal location (p < 0.001), followed by the left-side horizontal location (p = 0.020). The data suggests that gender is significantly related to the vertical and horizontal locations on both the right and left sides.

**Table 9:** Chi-square test for gender and mental foramen localization

Location	Chi-squared	p-value
Right-side vertical	7.083	0.029
Right-side horizontal	29.984	<0.001
Left-side vertical	7.315	0.026
Left-side horizontal	13.441	0.020

Statistically significant differences were found between gender and MF position in all locations (p < 0.05). Table 10 presents the results of chi-squared tests for the association between age and various location variables. The p-values for the right-side horizontal location (p = 0.010) and the left-side horizontal location (p = 0.053) are less than or close to 0.05, suggesting a significant association between age and these variables. However, the p-values for the right-side vertical (p = 0.143) and left-side vertical (p = 0.065) locations are greater than 0.05, indicating no significant association between age and these variables.

**Table 10:** Chi-square test for age group and mental foramen localization

Location	Chi-squared	p-value
Right-side vertical	12.188	0.143
Right-side horizontal	37.471	0.010
Left-side vertical	14.705	0.065
Left-side horizontal	31.199	0.053

No statistically significant association was found between age and vertical MF position on either side (p > 0.05). However, there was a significant relationship between age and horizontal location on the right side (p = 0.010) and a borderline significant association on the left side (p = 0.053), suggesting a tendency for more anterior positioning in older individuals.

## Discussion

This retrospective cross-sectional study analyzed the localization of the MF in 504 panoramic radiographs from an adult Saudi population. The MF is an important anatomical landmark that transmits the mental nerve and vessels [1]. Precise knowledge of its location has significant clinical implications for many dental procedures in the mandibular premolar region.

The most frequent vertical position was Zone B, aligned with the apices of the premolars on both the right and left sides. This corroborates past research by Ghandourah et al. and Mahabob et al., who also found the mental foramen most commonly situated inferior to the premolar's apices in Saudi populations (Al-Hasa region and multiple cities, respectively) [8,9]. The predominant horizontal location was Zone 4, between the first and second premolars, consistent bilaterally. This finding is supported by similar results from Turkish (Gungor et al.) and American (Phillips et al.) populations that localized the MF most frequently between mandibular premolars [5,11].

A key finding of this study was the high degree of bilateral symmetry, with concordant right and left MF positions in 87.5% of cases. This symmetry could be advantageous clinically in situations where the MF is difficult to visualize on one side. A clinician could presume a symmetrical location on the contralateral side with reasonable accuracy.

Regarding demographic factors, statistically significant differences were discernible in MF localization based on gender. This aligns with Chu et al., who also found significant gender variations in a Brazilian sample [16]. However, the current study did not find a statistically significant association between age and vertical MF position, although there was a tendency for more anterior horizontal positioning in older individuals. This contrasts with some previous studies that reported age-related changes in MF location [16,18]. The discrepancy may be due to differences in sample characteristics, age categorization, or statistical methods employed.

The standardization of horizontal and vertical zoning systems was a strength of this study, allowing comparison to other populations. The results provide directly relevant guidance on the highest risk areas for mental nerve injury during mandibular premolar procedures. In particular, the frequent MF location between premolars indicates a vulnerable zone requiring caution when administering anesthesia, placing implants, or performing surgery in this region [17,18].

However, some limitations exist. The convenience sampling methodology and restriction to a single city precludes generalization to the entire Saudi population. Further studies with larger, more diverse samples from multiple regions are warranted to elucidate potential geographical or age variations [8,9,18]. Cone-beam CT analysis could also reveal three-dimensional localization patterns not discernible on panoramic radiographs [9,17,18].

In conclusion, within the limitations of this retrospective panoramic study, the MF was most frequently located vertically below the apices of mandibular premolars (Zone B) and horizontally between the first and second premolars (Zone 4) in the Saudi adult population. A high degree of bilateral symmetry was observed. Significant variations occurred based on gender, with a tendency for more anterior horizontal positioning in older individuals. These findings guide the typical location of this vital anatomical structure to avoid neurovascular injury during dental procedures in the premolar area [8,17,18]. However, clinicians should remain vigilant for potential individual variations.

## Conclusions

This retrospective panoramic study on MF localization in an adult Saudi population found the MF was most frequently located vertically below the apices of mandibular premolars (Zone B) and horizontally between the first and second premolars (Zone 4), with high bilateral symmetry. Statistically significant gender differences were observed in vertical and horizontal positioning. No significant association existed between age and vertical position, though there was a non-significant tendency for more anterior horizontal positioning in older individuals. The standardized zoning system allowed precise localization and comparison across populations. The results guide clinicians on areas of neurovascular injury risk during dental procedures in the premolar region. Caution should be exercised, considering typical mental foramen location and individual variations. Further research with larger, diverse samples and advanced imaging is recommended to validate findings and explore potential geographical and age-related variations.
