# Correction: Panoramic Assessment of Mental Foramen Location in Adult Patients in Riyadh, Saudi Arabia: A Retrospective Cross-Sectional Study

**DOI:** 10.7759/cureus.c432

**Published:** 2026-05-13

**Authors:** Yahia Ahmad, Nadir Alajmy, Khalid Almobiedh, Abdulrahman Alsemari, Ghanem Alfailakawi, Soud Alenezi, Raviraj Jayam

**Affiliations:** 1 College of Dentistry Internship Department, Riyadh Elm University, Riyadh, SAU; 2 Oral and Maxillofacial Surgery and Diagnostic Sciences, Riyadh Elm University, Riyadh, SAU

The author list of this article has been corrected to include Raviraj Jayam as last author. Dr. Jayam was the lead supervisor and chief investigator of this work and was erroneously omitted from the original publication.

